# Impact of maternal body mass index on pregnancy outcomes among Indian women

**DOI:** 10.6026/9732063002001257

**Published:** 2024-10-31

**Authors:** Saloni Gandhi, Kishor Chauhan, Priyank Rajan, Mayur Wanjari, Labdhi Sangoi, Ravi Sangoi

**Affiliations:** 1 Department of Obstetrics and Gynaecology, Sumandeep Vidyapeeth University, Gujarat, India; 2 Department of Obstetrics and Gynaecology, Smt. B. K. Shah Medical Institute & Research Centre, Sumandeep Vidyapeeth University, Gujarat, India; 3 Department of Paediatric Haematology Oncology, Royal Bristol Hospital for Children, Bristol, United Kingdom; 4 Department of Research, Datta Meghe Institute of Higher Education & Research (DMIHER), Sawangi, Maharashtra, India; 5 Department of Research, Government Medical College, Jalna, Maharashtra, India; 6 Department of Internal Medicine, Government Medical College and General Hospital, Baramati, Pune, Maharashtra, India

**Keywords:** Maternal BMI, pregnancy outcomes, feto-maternal complications, caesarean section, pre-eclampsia, gestational diabetes, underweight

## Abstract

Maternal body mass index is a key factor that essentially regulates pregnancy outcome with respect to maternal and neonatal health. Maternal bodies, whether underweight or obese during pregnancy, can significantly increase the risk of adverse outcomes for both mothers and newborns. Therefore, it is of interest to evaluate the impact of maternal BMI on pregnancy outcomes and feto-maternal complications related to various BMI categories in a tertiary care setting. Hence, we recruited 250 pregnant women and divided them into five subgroups based on their BMI. We collected data on pregnancy complications, modes of delivery and maternal and neonatal outcomes. We performed tests of significance between categories of BMI and clinical outcomes. Percentage distribution by BMI: normal weight 49.2%, underweight 28.4%, overweight 15.6%, obese 6% and morbidly obese 0.8%. There was significant variability in higher BMI with incidences of caesarean section, pre-eclampsia, gestational diabetes, and NICU admissions. Anaemia rates were higher in underweight women, whereas pregnancy and childbirth-related complications like PPH and macrosomia were more pronounced in obese women. Severe extremes in BMI are associated with drastic adverse consequences, both for maternal and neonatal outcomes. Effective weight management is therefore key to achieving favourable pregnancy outcomes. Low BMI increases the risk of preterm birth and anaemia; high BMI raises the risk of gestational diabetes and hypertensive disorders. Key strategies include preconception counselling, tailored nutrition and physical activity.

## Background:

Indeed, body mass index is a really crucial measurement during pregnancy in order to indicate health risks. Adverse maternal and fetal outcomes have been associated with low weight and overweight. According to the WHO, malnutrition embraces disorders classified as underweight and overweight disorders, over nutrition being the later one; thus, they present great public health problems in almost all countries worldwide [1, 2]. Maternal BMI is also significantly influential on the outcome of a pregnancy. Pregnancy complications, including lower birth weights resulting in preterm babies and maternal anaemia, are likely to be experienced by women who were underweight prior to becoming pregnant [3]. Indeed, recurrent issues of food insecurity and inadequate prenatal care in developing countries mean that a high percentage of women in these countries remain at low weights [4, 5]. Overweight and obese women, on the other hand, are at higher risk for gestational diabetes mellitus, hypertensive disorders, pre-eclampsia and caesarean delivery. Additionally, infants born to overweight and obese mothers are more likely to experience adverse outcomes such as macrosomia and NICU admission [6, 7, 8- 9]. Under nutrition and obesity coexist among the rural and semi-urban areas in India. Recent estimates indicate that 23% of Indian women are underweight, while about 20% are obese or overweight. This reflects double malnutrition in their country, as revealed by recent trends [10, 11]. Rapid nutritional transitions and socioeconomic inequities tend to amplify this trend in nutritional deficiencies in developing countries. Some have limited access to nutritious food; others are increasingly adhering to a Westernized diet consisting of high-calorie, nutrient-deficient processed foods that contribute to obesity [12]. Today, it has become undeniable that increasing rates of obesity and its comorbidities in populations from the developing world, especially rural, are a point of concern [13]. Therefore, it is of interest to demonstrate how maternal nutrition influences pregnancy outcomes and offer valuable suggestions for health care interventions tailored to women with varying BMI levels.

## Materials and Methods:

This was a prospective observational study conducted in the Obstetrics and Gynaecology Department of Obstetrics and Gynaecology at District General Hospital Piparia from January 2019 to June 2020, following ethical approval. The study enrolled a total of 250 pregnant women with singleton pregnancies who presented during their first trimester. BMI was calculated using the Quetelet index (weight in kg/height in meters squared), and participants were categorized into underweight (<18.5), normal weight (18.5-24.9), overweight (25.0-29.9), obese (30.0-39.9), and morbidly obese (>40). Inclusion criteria included women in their first trimester, aged 18-40 years, with no pre-existing systemic diseases and the exclusion criteria comprised of women with multiple pregnancies, those presenting after the first trimester, and women with chronic medical conditions such as diabetes or cardiac diseases. Data on maternal demographics, BMI, pregnancy complications (*e.g.*, anaemia, GDM, pre-eclampsia), mode of delivery and neonatal outcomes (*e.g.*, birth weight, NICU (Neonatal Intensive Care Unit) admissions) were collected. Statistical tests included Kruskal-Wallis One-way ANOVA, chi-square tests and student unpaired t-tests. A p-value ≤ 0.05 was considered significant.

## Results:

Table 1 Distribution of Population Based on BMI. Table 2 presents the mean age of women in each BMI category and the percentage of women in the 21-25 years age group. The mean age increased with higher BMI categories, with the morbidly obese group having the highest mean age (31.50 years). Table 3 highlights the variation in pregnancy history among different BMI categories, with obese women more likely to be multigravida. Table 4 shows the distribution of pregnancy outcomes such as preterm, term, post-term deliveries and abortion rates across BMI categories. (Table 5) outlines the mode of delivery (vaginal vs. caesarean) in each BMI category. Vaginal deliveries were more common in the underweight and normal BMI groups, while caesarean sections were significantly more frequent in the obese (53.3%) and morbidly obese (50%) groups, highlighting the increased risk of surgical intervention with higher BMI. Table 6 lists NICU admissions and incidences of macrosomia by BMI category. Table 7 provides data on antepartum complications, including anaemia, pre-eclampsia, gestational diabetes mellitus (GDM) and antepartum haemorrhage (APH). Pre-eclampsia and GDM were significantly higher in the obese and overweight groups, with obesity being associated with a particularly high rate of GDM (26.67%). Table 8 highlights postpartum complications such as postpartum haemorrhage (PPH) and wound gaping.

## Discussion:

In this study, we aimed to assess the impact of maternal BMI on pregnancy outcomes in the Indian population. Our findings align with recent research that both low and high BMI levels significantly affect maternal and neonatal health. The data showed that being underweight or overweight during pregnancy is linked to a higher risk of problems during labour and delivery, including preeclampsia, gestational diabetes mellitus (GDM), caesarean sections (CS) and bad outcomes for the baby, such as macrosomia and NICU admissions. The majority of our study population had a normal BMI (49.2%), comparable to other studies in India that report normal BMI prevalence rates ranging from 40% to 60% [14]. However, the proportion of underweight women (28.4%) remains significant in rural populations, reflecting the continued prevalence of under nutrition in certain areas of India [15].

On the other hand, the nutritional transition and lifestyle changes in rural settings likely contribute to the increasing prevalence of obesity and overweight [16].

Underweight women were more likely to experience anaemia (23.94%), as seen in other studies evaluating maternal nutrition in developing countries [17]. Anaemia in pregnancy can lead to low birth weight and preterm delivery, both of which were observed at higher rates in this group [18]. Similarly, studies have shown that underweight women face a higher risk of preterm labour and low birth weight infants due to insufficient nutritional reserves [19]. On the other hand, overweight and obese women showed higher incidences of hypertensive disorders, such as pre-eclampsia (38.46% in overweight and 40% in obese women), which aligns with global trends [20]. A meta-analysis demonstrated that the risk of pre-eclampsia doubles with each 5-7 kg/m^2^ increase in BMI [21]. Insulin resistance, systemic inflammation and vascular dysfunction commonly observed in obese pregnant women contribute to this heightened risk [22]. Gestational diabetes was also notably higher in overweight (10.26%) and obese (26.67%) women [23]. This finding is consistent with recent research indicating that maternal obesity is a major risk factor for the development of GDM, with overweight women being three times more likely to develop the condition compared to women with normal BMI [24]. GDM not only increases the risk of macrosomia but also predisposes mothers to type 2 diabetes later in life [25]. One of the most significant findings of our study is the strong association between maternal BMI and mode of delivery. Caesarean sections were more common in obese women (53.3%) compared to those with normal BMI (30.9%) [26]. Various studies consistently report an increased rate of caesarean sections (CS) in overweight and obese women [27]. The reasons for this trend include fetal macrosomia, prolonged labour and dysfunctional uterine contractions in obese women [28]. Furthermore, literature suggests that obese women are more likely to have labour-induced and less likely to have a successful vaginal delivery, which further elevates CS rates [29]. Efforts to optimize maternal BMI through targeted interventions such as preconception counselling, weight management programs and regular prenatal care are crucial for improving pregnancy outcomes. Addressing both under nutrition and obesity among women of reproductive age can significantly reduce the burden of adverse maternal and neonatal outcomes. Public health policies should focus on increasing access to nutritional education and prenatal care in rural areas, promoting healthy weight gain during pregnancy and managing pre-existing nutritional deficits. By prioritizing maternal health, we can ensure better outcomes for both mothers and their babies, reducing the long-term impact of malnutrition on future generations [30]. The mechanism underlying this association is unclear and is worthy of further investigation [31].

## Conclusion:

The significant impact of maternal BMI on both maternal and neonatal outcomes in Indian population is of interest. Data shows that both underweight and obesity pose substantial risks during pregnancy, affecting maternal health through complications such as anaemia, pre-eclampsia and gestational diabetes. Extreme BMI also affects the outcome of delivery, with higher rates of caesarean sections and a higher risk of neonatal complications like macrosomia and NICU admissions.

## Figures and Tables

**Table 1:** Demonstrates distribution of the study participants across different BMI categories.

**BMI Category**	**No. of Women**	**% of Total**
Underweight	71	28.40%
Normal	123	49.20%
Overweight	39	15.60%
Obese	15	6.00%
Morbidly Obese	2	0.80%

**Table 2:** Mean age and age group distribution by BMI

**BMI Category**	**Mean Age (years)**	**Age Group (21-25 years)%**
Underweight	23.83	47.89%
Normal	24.65	34.96%
Overweight	24.59	51.28%
Obese	25.33	33.33%
Morbidly Obese	31.5	-

**Table 3:** Gravida status by BMI

**BMI Category**	**Primigravida (%)**	**Multigravida (%)**
Underweight	45.07%	54.93%
Normal	47.15%	52.85%
Overweight	53.85%	46.15%
Obese	26.67%	73.33%

**Table 4:** Pregnancy outcomes by BMI

**BMI Category**	**Preterm (%)**	**Term (%)**	**Post-term (%)**	**Abortion (%)**
Underweight	14.08%	71.80%	11.20%	2.82%
Normal	14.63%	74.79%	9.75%	0.81%
Overweight	15.38%	64.10%	12.80%	7.69%
Obese	13.33%	60.00%	13.33%	13.33%

**Table 5:** Mode of delivery by BMI

**BMI Category**	**Vaginal (%)**	**Caesarean (%)**
Underweight	71.80%	25.40%
Normal	68.30%	30.90%
Overweight	51.30%	41.00%
Obese	33.30%	53.30%
Morbidly obese	-	50.00%

**Table 6:** NICU admissions and neonatal complications by BMI

**BMI Category**	**NICU Admissions (%)**	**Macrosomia (%)**
Underweight	2.80%	-
Normal	3.30%	2.46%
Overweight	20.50%	11.11%
Obese	26.70%	15.38%

**Table 7:** Antepartum complications by BMI

**BMI Category**	**Anaemia (%)**	**Pre-eclampsia (%)**	**GDM (%)**	**APH (%)**
Underweight	23.94%	21.13%	2.82%	4.23%
Normal	5.69%	22.76%	3.25%	4.88%
Overweight	7.69%	38.46%	10.26%	10.26%
Obese	6.67%	40.00%	26.67%	13.33%

**Table 8:** Postpartum complications by BMI

**BMI category**	**PPH (%)**	**Wound gaping (%)**	**Total complications (%)**
Underweight	2.82%	8.45%	15.50%
Normal	4.07%	4.07%	10.60%
Overweight	15.38%	15.38%	43.60%
Obese	20		
